# Liquid-Liquid Phase Separation of TDP-43 and FUS in Physiology and Pathology of Neurodegenerative Diseases

**DOI:** 10.3389/fmolb.2022.826719

**Published:** 2022-02-02

**Authors:** Jenny L. Carey, Lin Guo

**Affiliations:** Department of Biochemistry and Molecular Biology, Thomas Jefferson University, Philadelphia, PA, United States

**Keywords:** liquid-liquid phase separation, FUS, TDP-43, stress granules, ALS

## Abstract

Liquid-liquid phase separation of RNA-binding proteins mediates the formation of numerous membraneless organelles with essential cellular function. However, aberrant phase transition of these proteins leads to the formation of insoluble protein aggregates, which are pathological hallmarks of neurodegenerative diseases including ALS and FTD. TDP-43 and FUS are two such RNA-binding proteins that mislocalize and aggregate in patients of ALS and FTD. They have similar domain structures that provide multivalent interactions driving their phase separation *in vitro* and in the cellular environment. In this article, we review the factors that mediate and regulate phase separation of TDP-43 and FUS. We also review evidences that connect the phase separation property of TDP-43 and FUS to their functional roles in cells. Aberrant phase transition of TDP-43 and FUS leads to protein aggregation and disrupts their regular cell function. Therefore, restoration of functional protein phase of TDP-43 and FUS could be beneficial for neuronal cells. We discuss possible mechanisms for TDP-43 and FUS aberrant phase transition and aggregation while reviewing the methods that are currently being explored as potential therapeutic strategies to mitigate aberrant phase transition and aggregation of TDP-43 and FUS.

## Introduction

Within the past decade, there has been an exponential rise in studies investigating the role of liquid-liquid phase separation (LLPS) in neurobiology and its pathological consequences in neurodegenerative diseases, such as amyotrophic lateral sclerosis (ALS) and frontotemporal dementia (FTD). It is now understood that LLPS is a universal mechanism of condensing proteins and/or RNAs into liquid-like foci for numerous functions. In neurons, RNA and protein foci have been reported in various settings including DNA damage repair, local mRNA translation, and neuronal stress response. Overwhelmingly, proteins involved in this phenomenon are RNA-binding proteins (RBPs) and many are implicated in ALS and FTD. This review will focus on two specific RNA-binding proteins implicated in ALS and FTD pathology: TAR DNA-binding protein 43 (TDP-43) and fused in sarcoma/translocated in liposarcoma (FUS/TLS). Their propensity to undergo LLPS has been well-characterized and they serve as model proteins to study the dynamics of protein phase separation in relation to neurodegenerative diseases.

## RNA-Binding Proteins Implicated in ALS and FTD

ALS is a fatal motor neuron disease that affects both upper and lower motor neurons and leads to their eventual degeneration whereas FTD is another neurodegenerative disease that affects frontal and temporal cortex. Proteinopathies implicated in both conditions overlap, including TDP-43 and FUS aggregation in the respective neuronal populations (Ling et al., 2013). Other RBPs implicated in these diseases include heterogeneous nuclear ribonucleoprotein A1 (hnRNP A1), and heterogeneous nuclear ribonucleoprotein A2/B1 (hnRNP A2/B1) amongst the FET protein family members (FUS, EWSR1 and TAF15) and others; however, we will focus broadly on the roles of TDP-43 and FUS phase separation in physiological and pathological neuronal states. The significance of these overlapping proteinopathies is not well-characterized, but the RBPs involved share many structural and functional similarities, which will be discussed in the following sections.

### Structure of TDP-43 and FUS

RNA-binding proteins are a class of proteins that interact with RNA and largely function in transcriptional and translational regulation. Many of these proteins are conserved from yeast to mammals and contain regions of low complexity (Hughes et al., 2021). While low sequence complexity imparts functional benefits to these proteins, it often proves difficult to study the full-length structure of RBPs. Previously, fragments of these proteins were studied alone or in complex with binding partners. However, with the advent of cryo-EM and other modified techniques, we can deduce more about the full-length structure of these proteins.

**FIGURE 1:**
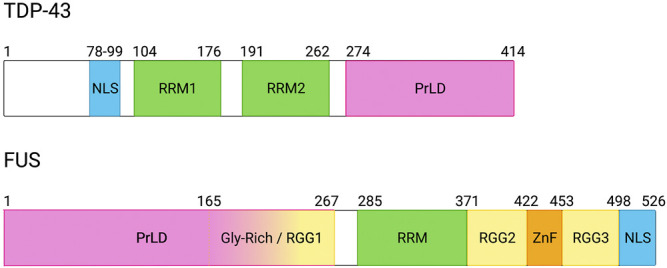
Domain architecture of TDP-43 and FUS. TDP-43 and FUS contain similar structural domains with some notable differences. Both contain a prion-like domain (PrLD), which is characterized by a high concentration of glycine, glutamine, asparagine, and tyrosine amino acids with low sequence complexity; however, the TDP-43 PrLD contains one tyrosine residue whereas the FUS PrLD is comprised of multiple tyrosines that can be phosphorylated. Additionally, the FUS PrLD overlaps with an arginine-glycine-rich (RGG) domain between residues 165 and 267. Notably, FUS contains multiple RGG domains throughout its structure in addition to a zinc-finger motif (ZnF). Both TDP-43 and FUS contain nuclear localization signals (NLS), although they differ in sub-classifications and subsequent nuclear import receptor interactions. Both proteins also contain RNA recognition motifs (RRMs), which are important domains for RNA-binding. Created with BioRender.com.

The N-terminal half of FUS contains a PrLD (residues 1-267), which are low complexity domains enriched for asparagine, glutamine, tyrosine and glycine residues and similar in amino acid composition to yeast PrLDs (prion-like domains; PrLDs) (March et al., 2016). The C-terminal region of the PrLD (residues 165-267) are enriched for glycine and is sometimes documented as a distinct arginine-glycine-glycine (RGG) domain (RGG1). The FUS RRM spans residues 285-371 and is flanked by a N-terminal glycine-rich domain (Gly-rich/RGG1) and a C-terminal RGG region (RGG2). FUS contains additional RGG domains throughout its primary structure. These regions of low-complexity surround a zinc-finger domain (ZnF) near the C-terminus (Figure 1). The ZnF domain is important for double-stranded nucleotide binding and has recently been implicated to facilitate a bipartite RNA recognition domain, along with the RRM and RGG domain (Loughlin et al., 2019). At its C-terminus, FUS contains a PY-NLS, which directs nuclear import through binding to Kapb2.

TDP-43 N-terminal region harbors a classical nuclear localization signal (NLS). Afterwards, TDP-43 contains two RNA recognition motifs (RRMs) near the middle of the protein that are separated by a short, 15-nucleotide sequence (Figure 1). Additionally, TDP-43 contains a C-terminal prion-like domain (PrLD). However, the TDP-43 PrLD only contains a single tyrosine, which is in stark contrast to other RBPs such as FUS, which contains multiple tyrosine residues within its PrLD.

### Function of TDP-43 and FUS

RNA-binding proteins have active roles in RNA metabolism and splicing in neurons, and contribute to normal neuronal physiology (Polymenidou et al., 2011; Rogelj et al., 2012; Masuda et al., 2016). One such family of RBPs that are involved in RNA splicing include the heterogenous nuclear ribonucleoprotein (hnRNP) family. Notable members include hnRNPA1, hnRNPA2/B1, and FUS. These proteins were originally identified through a crosslinking study to RNA and have since been implicated in neurodegenerative diseases. Another RBP that has important functional roles in neurons is TDP-43. While its exact function remains elusive, TDP-43 is implicated in multiple neurodegenerative diseases, suggesting it plays an important role in normal neuronal physiology.

In mammalian neurons, TDP-43 has been shown to localize to transcriptionally-active sites, including the nucleolus, suggesting it plays a role in mRNA processing (Casafont et al., 2009). In healthy cells, TDP-43 plays a large role in cryptic exon repression of exons with UG-rich domains and loss of TDP-43-mediated regulation is associated with cell death via nonsense-mediated decay in mouse embryonic stem cells (Ling et al., 2015). Specific targets include *ATG4B* and *RANBP*, which are involved in autophagy and nuclear import, thus potentially play a role in TDP-43 mislocalization to and accumulation in the cytoplasm. TDP-43 facilitates mRNA granule axonal tracking in fly motor neurons and cultured primary embryonic mouse motor neurons to promote synapse-localized translation (Alami et al., 2014; Briese et al., 2020). TDP-43 is also implicated in physiological structure of neurons including dendritic spine and synapse formation. One study in cultured prenatal rat hippocampal and cortical neurons demonstrates an interesting phenomenon where both TDP-43 overexpression and knockdown reduced dendritic complexity (Herzog et al., 2017). A follow-up study identified that TDP-43 achieves this effect by inhibiting CREB activation and that restoration of CREB signaling rescued dendritic phenotype in cultured embryonic rat hippocampal neurons (Herzog et al., 2020). TDP-43 is also implicated in mitochondrial regulation. In a study expressing human TDP-43 in mouse neurons, mitochondria clustered in motor neurons, suggesting TDP-43 may function in mitochondrial trafficking (Shan et al., 2010). At the neuromuscular junction (NMJ), TDP-43 seems to be integral to NMJ formation and maintenance (Campanari et al., 2021; Strah et al., 2020). It is possible TDP-43 is involved in these important structures thorough a relationship with cytoskeletal proteins, where TDP-43 seems to be important for proteins involved in synaptic microtubule organization and transcription of microtubule associated protein 1B (MAP1B) (Godena et al., 2011). To this end, many investigators have recently focused on the role of TDP-43 in splicing the Stathmin-2 (STMN2) gene. Stathmin-2 is a protein involved in microtubules and is essential for axonal regeneration; however, loss of functional TDP-43 is associated with premature polyadenylation of STMN2 pre-mRNA and subsequent loss of the mRNA transcripts and STMN2 protein (Klim et al., 2019; Melamed et al., 2019).

FUS also has important roles outside the nucleus in neurons. Like TDP-43, FUS is involved in dendritic maturation and complexity of mouse hippocampal neurons via transporting mRNA to the dendrites (Fujii and Takumi, 2005). In rat hippocampal axons, FUS is demonstrated to regulate microtubule growth by promoting trafficking of *Ddr2* RNA and this function was determined to be phase-dependent, where neurons expressing phase-separated FUS had mislocalized RNAs (Yasuda et al., 2017). Previous studies have reported FUS localization to the synapses of hiPCS and rat motor neurons (Deshpande et al., 2019) in addition to rat hippocampal neurons (Schoen et al., 2015). Interestingly, in a recent publication, FUS was demonstrated to regulate DNA replication, and a sequencing experiment on U2OS cells with knockdown FUS, many FUS-dependent replication domains were mapped to have function at synapses (Jia et al., 2021), suggesting FUS may influence the physiology of synapses. Previous reports may support this function, including studies where FUS was demonstrated to regulate synaptic transmission in *Drosophila* (Machamer et al., 2014) mediate acetylcholine receptor transcription in mice (Picchiarelli et al., 2019).

Both TDP-43 and FUS have been demonstrated to undergo LLPS *in vitro* (Li et al., 2018; Molliex et al., 2015; Patel et al., 2015). LLPS is a demixing process in which a homogeneous solution is separated into two coexisting liquid phases: a high concentration condensed phase and a low concentration dilute phase. Evidences exist that certain functions of TDP-43 and FUS are closely related to their ability to phase-separate. For example, in a recent publication, Hallegger et al. showed TDP-43 mutants with different condensation propensities bind to different and specific RNA regions across the transcriptome, and as a result, affects its RNA processing functions (Hallegger et al., 2021). Moreover, a mouse model expressing LLPS-deficient TDP-43 demonstrated impaired neuronal function and translational regulation (Gao et al., 2021). In the next sections, we will review the factors that mediate LLPS of TDP-43 and FUS and cellular functions that are related to their phase separation property. While LLPS does not necessarily imply a phase transition (e.g., from liquid to solid), phase separation can involve aberrant phase transition from liquid state to solid state. Aberrant phase transition of TDP-43 and FUS is implicated in ALS and FTD, therefore, we will also review strategies to prevent and reverse their aberrant phase transition. For a more detailed review on the roles played by disturbed liquid-liquid phase separation of RNA-binding protein in the cellular pathology of ALS, we refer to the review by Milicevic et al. within this Special Issue.

## Factors That Mediate Liquid-Liquid Phase Separation of TDP-43 and FUS

Liquid-liquid phase separation (LLPS) is a process by which cells can sequester biomolecules into membraneless organelles. First demonstrated in P granules (Brangwynne et al., 2009), membraneless organelles are dynamic and demonstrate characteristic physical properties of Newtonian liquid droplets, including: spherical shape, fusion and fission, and response to shear flow [further reviewed in (Hyman et al., 2014)]. Another property of liquid condensates is their reversible assembly. Membraneless organelles can quickly form via LLPS and the foci likewise disperse once the surrounding conditions change. A specific subset of membraneless organelles are ribonucleoprotein (RNP) granules that specifically incorporate RNA and RNA-binding proteins into liquid droplets. Each RNP granule has a unique composition and forms in response to different stimuli, thus, creating a vast network of phase-separating proteins. Proteins involved in membraneless organelles are commonly referred to as scaffold/driver, regulator, or client proteins. Scaffold proteins are the key drivers of granule nucleation and are multivalent while client proteins get recruited to phase-separate after nucleation and have lower valency (Banani et al., 2016). Regulators are generally molecules that can induce posttranslational modifications and affect the affinity of scaffold molecules for itself and/or clients, thus affecting LLPS of proteins and formation of membraneless organelles; however, a recent study identified a multitude of other regulators for membraneless organelles (Berchtold et al., 2018). Many proteins can undergo LLPS, but a large family of proteins known to undergo LLPS are RNA-binding proteins (RBPs). These proteins contain many classical structural and biophysical properties that facilitate LLPS, which will be discussed in the following section.

### Multivalency

The valency of a protein relates to the amount of interactions it can make with other biomolecules. Most RBPs are afforded multivalency through multiple RNA- and DNA-binding domains. For instance, multivalency allows RBPs to act as a connection between other proteins in transcriptional regulation complexes and the target RNAs that are being post-transcriptionally modified. In regards to LLPS, multivalency is a key driver of protein phase separation. In an artificial system, Li et al. demonstrates an association between multivalency and LLPS propensity (Li et al., 2012). Specifically, they utilized proteins with SH3 domains and proline-rich motifs (PRMs) to show that increasing the number of SH3 binding domains and the amount of available PRMs also increases LLPS (Li et al., 2012). It is clear that multivalency is a prevailing property related to protein phase separation. Indeed, many other factors that mediate LLPS work by adding valency to the protein of interest, including RNA-binding domains and intrinsically disordered regions.

### RNA-Binding Domains

A near-ubiquitous motif found in RNA-binding proteins is the RNA recognition motif (RRM), which adopts a secondary structure and facilitates diverse single-stranded nucleic acid binding (Maris et al., 2005). This domain is also termed the RNA-binding domain (RBD) or ribonucleoprotein domain (RNP) and is a roughly 90 residue sequence that is positively charged and consisting of aromatic residues (Adam et al., 1986; Swanson et al., 1987). The flexibility of sequence homology in this region has led to the discovery of multiple RRM structures. The first RRM structure studied was that of U1 small nuclear ribonucleoprotein A (U1A). Structural analyses determined the RRM folded into an alpha-beta sandwich with four-stranded antiparallel beta-sheets in the middle and two alpha-helices acting as the “bread” on either side (Nagai et al., 1990). Since this discovery, multiple RRMs have been identified and characterized. RRMs and other RNA-binding motifs are key modulators of phase separation of proteins such as TDP-43 and FUS because they increase the protein valency when RNA is nearby. Moreover, since RNA is a major component of RNP granules and is present throughout the cellular environment, RBPs like TDP-43 and FUS are constantly interacting with RNA.

As mentioned above, RNA-binding domains, including RRMs, promote RBP multivalency and thus RNA-binding can regulate the LLPS dynamics of its protein interactors. For instance, Grese et al. demonstrated a sequence- and length-specific effect of RNA on TDP-43 LLPS, where GU RNA oligonucleotides induced large droplets *in vitro* and longer GU RNA oligos induced even larger droplets, suggesting RNA can induce LLPS (Grese et al., 2021). This effect is not unique to TDP-43, and multiple investigations have demonstrated that RNA can also regulate FUS LLPS. In 2013, Schwartz et al. demonstrated that prD RNA could promote FUS assemblies in solution at lower concentrations than full-length FUS alone, and that RNA in higher concentrations could inhibit FUS assembly (Schwartz et al., 2013), which is consistent with a later study that also demonstrated this effect was specific to FUS RNA-binding domains and not the PrLD domain (Burke et al., 2015).

While RRMs are present in most RBPs, other RNA-binding motifs include zinc finger (ZnF) domains and RGG domains. FUS domain structure includes both motifs (Figure 1), and each has similar RNA-binding affinities (Schwartz et al., 2013). Previous studies demonstrated both these domains facilitate FUS stress granule recruitment (Bentmann et al., 2012), suggesting these domains play an important role in FUS LLPS. Interestingly, a recent study published by Loughlin et al. demonstrated cooperative binding between the ZnF and RGG domains whereby the ZnF can recognize RNA sequence and the RGG domain can remain unstructured to facilitate RNA structure recognition by the adjacent RRM (Loughlin et al., 2019).

### Intrinsically Disordered Regions

Intrinsically disordered regions are a hallmark of phase-separating proteins. Intrinsically disordered regions (IDRs) are aptly named due to their lack of secondary or higher-order structure. This disordered “structure” is often imparted by large stretches of low sequence complexity, which generally include high concentrations of polar and aromatic residues, including arginines and tyrosines. A specific sub-type of intrinsically disordered regions are prion-like domains (PrLDs) which are similar in composition to yeast prions and drive protein phase separation (Franzmann et al., 2018). Multiple investigators have demonstrated that these low-complexity domains are necessary and sufficient for protein phase separation (Burke et al., 2015; Kato et al., 2012; Kato and McKnight, 2021; Lin et al., 2015; Molliex et al., 2015), and this behavior is often due to cation- π and π - π stacking interactions imparted by aromatic residues (Bogaert et al., 2018; Lin et al., 2017). However, the molecular dynamics of PrLD LLPS can be influenced by other disordered domains within the protein. For example, J. Wang et al. demonstrated the PrLD of FUS is sufficient to undergo LLPS, yet its propensity to phase-separate was enhanced in constructs where the RGG domain was present, suggesting that other IDRs can enhance protein LLPS (Wang J. et al., 2018).

RGG domains were initially called glycine-arginine-rich (GAR) domains due to their high concentrations of arginine and glycine residues, and while not all RBPs have these domains, they are a common sequence motif found in most RBPs. Generally, these domains are recognized as stretches of arginine-glycine-glycine residues with less than four residues between the RGG motifs. These domains impart flexibility and low-complexity to the protein and have various functions including: cellular localization, nucleic acid binding, and protein-protein interactions [further reviewed in (Thandapani et al., 2013)].

In recent years, the inter- and intra-molecular interactions afforded by IDRs have been described in a “Spacer and Sticker” model, where intrinsically disordered regions assemble into a pseudo-structure of spacers and stickers. The stickers will adopt a local structure to facilitate intra- or inter-molecular interactions whereas the spacers are flexible regions between the stickers. The alternation between a semi-structured binding domain and a flexible linker imparts additional valency to the protein and the conformational flexibility needed to interact with surrounding biomolecules. Spacers and stickers also influence the phase-separation behavior of RBPs by facilitating weak intermolecular and intramolecular interactions. In general, the arginine- and/or tyrosine-containing stickers define the saturation concentration of the protein whereas the spacers can modulate the droplet dynamics after phase separation (Halfmann et al., 2011; Wang J. et al., 2018). In a specific example in hnRNPA1, Martin et al. demonstrated the aromatic residues of stickers can impart additional valency to the protein, thus regulating its phase-separating dynamics (Martin et al., 2020). Of note, this study also observed the aromatic patterning within the hnRNPA1 sequence can determine whether the condensate will remain liquid-like or mature into aggregates (Martin et al., 2020).

The FUS spacer and sticker interactions are well-characterized and highlight the contributions of RGG and PrLD interactions in phase separation. Wang et al. demonstrated the arginine from the C-terminal FUS RGG and the tyrosine from its N-terminal PrLD interact to drive LLPS (Wang J. et al., 2018). This observation is preceded by an *in vitro* study by Lin et al. that characterized the contributions of tyrosine to FUS LLPS (Lin et al., 2017). Both articles corroborate an earlier study by Kato et al. where they substituted tyrosine residues in the FUS PrLD for serines and observed decreased FUS stress granule recruitment in U2OS cells, demonstrating lack of tyrosine residues is associated with decreased phase separation (Kato et al., 2012). Another study by Bogaert et al. confirms the tyrosine-arginine interaction between the PrLD and C-terminal RGG domains, respectively, and that this interaction drives phase separation (Bogaert et al., 2018). Interestingly, a recent study by Dasmeh and Wagner investigated the evolution of stickers and spacers in FUS, and determined the tyrosine residues within the FUS PrLD were conserved while the rest of the PrLD evolved, suggesting the biological importance of these sticker residues in FUS phase separation (Dasmeh and Wagner, 2021). TDP-43 does not have the same domain architecture as FUS, and importantly does not harbor any RGG domains. However, lack of RGG domain does not prevent spacer and sticker interactions within TDP-43. Instead, Schmidt et al. recently identified sequence patterning of hydrophobic and hydrophilic motifs within the PrLD that regulate its phase separation (Schmidt et al., 2019). Similar to FUS, this study demonstrated that the aromatic residues within the hydrophobic segments drive TDP-43 phase separation; however, the precise location of these residues in relation to the hydrophilic conserved region was important (Schmidt et al., 2019).

### LARKS

Low-complexity, amyloid-like, reversible, kinked segments (LARKS) are another recently-described structural motif found in nuclear RBPs that can mediate phase separation of these proteins. LARKS were first determined in the low-complexity domains of FUS, hnRNPA1, and nup98 (Hughes et al., 2018). It was proposed that these LARKS act as Velcro to provide adhesion between LCDs to form phase-separated condensates. On the other hand, steric-zippers act as molecular glue to fasten together amyloidogenic segments to form amyloid fibrils. As a result, amyloid fibrils are irreversible while LARKS are reversible structures. LARKS may assemble the core of some FUS labile fibrils (Murray et al., 2017). Typically, LARKS are found within the low-complexity domain of proteins, and a recent study suggests LARKS are enriched in many proteins that undergo liquid-liquid phase separation across multiple species, including FUS and TDP-43 (Hughes et al., 2021). The significance of LARKS in the low-complexity domain is not fully understood, but previous work has demonstrated that LARKS affect the amyloid structure of proteins harboring LARKS by forming a kinked structure compared to amyloid steric zipper, and LARKS may contribute to hydrogel formation. These structures may thus explain the reversibility of the structures formed by LARKS (Hughes et al., 2018), in contrast to the irreversible structure of amyloid fibrils. In TDP-43, four segments within the LCD form LARKS, while six other segments from the LCD form steric zippers characteristic of spines of pathogenic amyloid fibrils (Guenther et al., 2018). Moreover, familial TDP-43 ALS variants convert LARKS to irreversible aggregates, providing a possible mechanism of aberrant phase transition from functional membraneless organelles to pathogenic amyloids (Guenther et al., 2018).

### Oligomerization Domains in RNA-Binding Proteins

Oligomerization domains can also contribute to the multivalency that drives phase separation. For example, the N-terminal domain (NTD) of TDP-43 can form oligomers when purified alone. However, the oligomerization domain itself does not aggregate or phase-separate. Instead, the weak interactions between the C-terminal PrLD in TDP-43 mediate its LLPS. However, TDP-43 NTD oligomerization can enhance the multivalency and as a result, enhancing phase separation of full-length TDP-43 (Wang A. et al., 2018). This enhancement is reduced when the oligomerization of NTD is disrupted by phosphomimic mutant S48E in the NTD (Wang A. et al., 2018). In addition to the N-terminal oligomerization domain, the short 321–340 region in the C-terminal domain (CTD) can also self-interact and form an α-helix upon self-assembly (Conicella et al., 2016; Masuda et al., 2016). Oligomerization of this short region is essential for TDP-43 phase separation, since ALS mutations within this region that disrupt self-interaction alter phase separation (Conicella et al., 2016).

### Post-Translational Modifications

Polar, uncharged residues typically constitute intrinsically-disordered regions and allow for weak interactions that facilitate LLPS. As such, disruption of these weak, electrostatic interactions through posttranslational modifications (PTMs), can affect protein phase separation (Webber et al., 2020). TDP-43 is unique in that a pathological hallmark of patient-derived TDP-43 aggregates is phosphorylated TDP-43 (Hasegawa et al., 2008). This suggests that post-translational modification of TDP-43 can affect its phase separation and aggregation. Multiple labs have investigated this topic, and Wang et al. found that a single phosphomimetic substitution in the N-terminal domain (S48E) disrupts TDP-43 phase separation and oligomerization *in vitro* (Wang A. et al., 2018). Others have found that TDP-43 acetylation can regulate its phase separation. For example, Cohen et al. identified multiple lysine acetylation sites in TDP-43 and used acetylation mimetic and acetylation-null mutants to demonstrate acetylation is associated with increased aggregation (Cohen et al., 2015). Another PTM of interest is TDP-43 ubiquitination because TDP-43 aggregates are typically ubiquitinated (Arai et al., 2006; Neumann et al., 2006). Hans et al. later identified multiple ubiquitination sites on TDP-43, and a subsequent paper demonstrated the ALS-associated mutation K263E is hyper-ubiquitinated (Hans et al., 2014; Hans et al., 2018). Overall, the association between TDP-43 post-translational modification and LLPS is not well-characterized, but would be interesting to pursue further considering the effects of post-translational modifications on TDP-43 aggregation.

The PTM landscape of FUS has been well-characterized, and a recent review by Rhoads et al. describes a multitude of residues that can be post-translationally modified (Rhoads S. et al., 2018). In general, PTMs, such as serine phosphorylation and arginine methylation, in the N-terminal PrLD disrupt FUS LLPS (Monahan et al., 2017; Rhoads S. N. et al., 2018; Hofweber and Dormann, 2019; Owen et al., 2020).

## Functional Role of Phase Separation of TDP-43 and FUS

**FIGURE 2:**
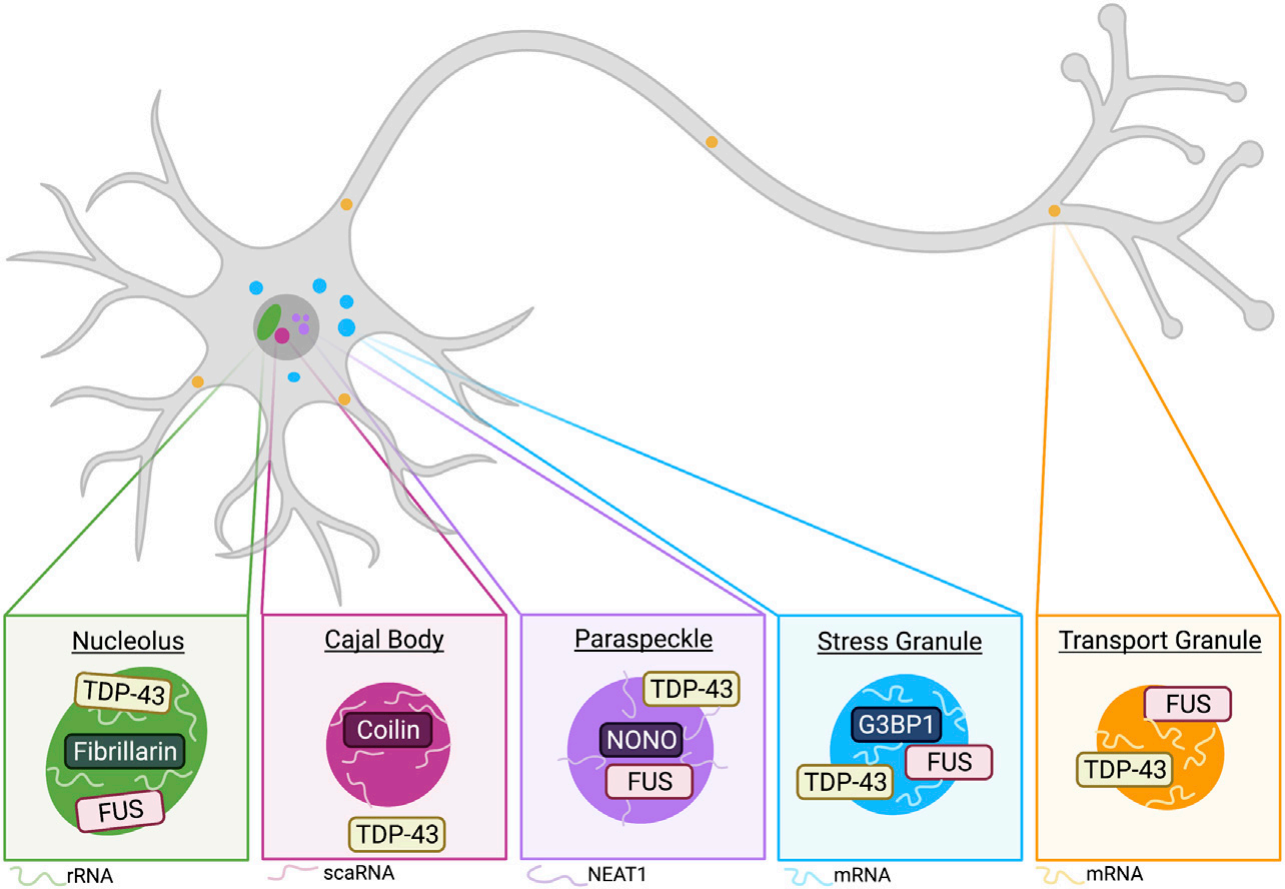
Phase-separated membraneless organelles have distinct components and functions. Schematic of membraneless organelles implicated in TDP-43 and/or FUS biology. Neurons contain nucleoli, which are condensed regions associated with ribosomal RNA synthesis. TDP-43 and FUS have been observed to localize to the nucleolus, which is commonly marked by fibrillarin and rRNA (see *Nucleoli*). Cajal bodies (CBs) are closely associated with nucleoli and commonly found in neurons. Major CB markers include coilin and the presence of small Cajal body-specific RNA (scaRNA). While not demonstrated to colocalize, TDP-43 is implicated in CB formation and regulation (see *Cajal Bodies*). Another nuclear membraneless organelle relevant to TDP-43 and FUS biology includes paraspeckles (see *Paraspeckles*). The long non-coding RNA (lncRNA) NEAT1 is an integral RNA component of paraspeckle organization, and these condensates are commonly marked by NONO. TDP-43 has been demonstrated to be important regulator of paraspeckle formation and it is shown to localize to the shell of paraspeckles, whereas FUS is generally localized to the core (West et al., 2016). Cytoplasmic stress granules are recently implicated in neurodegenerative diseases, and G3BP1 has been demonstrated as a central protein involved in SG formation (Yang et al., 2020). Stress granules stall protein translation and mRNA is a common RNA component of these condensates. Both TDP-43 and FUS demonstrate SG recruitment in neurons (see *Stress Response*). A final membraneless organelle covered in this review includes transport granules. These granules are observed in dendrites and axons of neurons. TDP-43 and FUS are both implicated in their formation, and these condensates often include mRNA, which is shuttled away from the soma for local translation (see *Transport Granules and Local Translation*). Created with BioRender.com.

As the molecular grammar governing the phase separation of RNA-binding proteins is becoming better understood, we are also gaining deeper insights into the *in vivo* physiological role of phase separation of these proteins. Liquid-liquid phase separation mediates the formation of biomolecular condensates that are found throughout eukaryotic cells, such as nucleoli in the nucleus, stress granules in the cytoplasm, and synaptic densities at the membrane. These biomolecular condensates can function at the molecular level by accelerating biochemical reactions via increasing local enzyme and substrate concentration or by inhibiting their activity through sequestration. They can also function at the mesoscale level by organizing cellular processes within cells including the DNA damage response cascade. Biomolecular condensates can also function at the cellular level by sensing rapid changes in environmental conditions and by triggering appropriate homeostatic responses. ALS/FTD-associated RNA-binding proteins including TDP-43, FUS, hnRNPA1, hnRNPA2, and TIA1 have been identified in multiple biomolecular condensates, and the phase separation of these RNA-binding proteins are important for the formation and function of these biomolecular condensates. Indeed, many cellular functions of these RNA-binding proteins depends on their phase separation. In this section, we will discuss the physiological roles played by these RNA-binding proteins in different biomolecular condensates (illustrated in Figure 2) and the cellular processes that depend on their phase separation.

### Nucleoli

Nucleoli are ubiquitous membraneless organelles that form via LLPS and are the location of ribosomal RNA synthesis and ribosome biogenesis in cells (Lafontaine et al., 2021). In primary human fibroblasts and human spinal motor neurons, FUS and TDP-43 are observed to localize to the nucleolus in response to DNA damage and transcriptional stress (Martinez-Macias et al., 2019). Interestingly, FUS nucleolar localization is not affected by the ALS-associated P525L mutation (Martinez-Macias et al., 2019). While their roles within nucleoli remains unclear, their localization to these membraneless organelles suggests some physiological function.

### Paraspeckles

Paraspeckles are another nuclear-localized membraneless organelle characterized by the presence of the long non-coding RNA NEAT1 (Shelkovnikova et al., 2014; An et al., 2018). It is shown that cross-regulation between NEAT1 and TDP-43 is essential for their functions in promoting states of pluripotency and differentiation in stem cells (Modic et al., 2019). Of note, healthy motor neurons typically do not form paraspeckles because they do not express the inducible NEAT1 isoform, NEAT1_2; however, a recent study by Grosch et al. demonstrated approximately two paraspeckles per motor neuron nucleus under normal conditions (Grosch et al., 2020). While paraspeckle formation has been documented in healthy motor neurons, multiple studies have demonstrated paraspeckle formation is more prevalent in ALS cases (Nishimoto et al., 2013; Shelkovnikova et al., 2018; An et al., 2019; Tyzack et al., 2020). FUS is required for formation of paraspeckles in HeLa cells (Hennig et al., 2015) and interacts with paraspeckle-associated protein NONO in SH-SY5Y cells (Shelkovnikova et al., 2014), suggesting FUS may play an integral role in paraspeckle function. Additionally, FUS is localized to the core of paraspeckles while TDP-43 is in the outer shell (West et al., 2016). Moreover, in ALS patients with GGGGCC (G4C2) expansion mutation in the *C9ORF72* gene, nuclear RNA foci formed by (G4C2)n colocalize with paraspeckle proteins such as FUS, highlighting the involvement of paraspeckle in ALS (Bajc Česnik et al., 2019).

### Cajal Bodies

Cajal bodies were first described as accessory bodies by Cajal and are distinct condensates found within the nucleus of neurons and other cells that undergo high rates of transcription (Sawyer et al., 2016; Lafarga et al., 2017). While TDP-43 is not localized to SMN-positive Cajal Bodies in rat dorsal root ganglia (Casafont et al., 2009), a recent study suggests TDP-43 plays a role in their formation and regulation via trafficking small Cajal body-specific RNA (scaRNA) to the Cajal bodies (Izumikawa et al., 2019). Additionally, human TDP-43 expression in transgenic mice displayed increased number of gemini of Cajal bodies (GEMs) (Shan et al., 2010), indicating TDP-43 may regulate formation of these nuclear membraneless organelles.

### Stress Response

Neuronal cells often encounter various forms of cellular stress, and one way in which neurons respond to this stress is through the formation of transient stress granules, which sequester untranslated mRNAs and associated proteins in an effort to decrease energy demands until the stressor is removed from the system. These granules are commonly induced by heat, osmotic and oxidative stress, and it has been shown that each stressor induces unique networks of stress granule proteins and RNAs (Maxwell et al., 2021). A major coordinator of stress granules is G3BP1, which is a scaffold stress granule protein (Yang et al., 2020). Another protein at the core of the cellular stress response is phosphorylated eIF2a, which results in overall decrease in protein synthesis (Harding et al., 2003; Pakos-Zebrucka et al., 2016; Boye and Grallert, 2020) and is regulated by TIA1 (Meyer et al., 2018).

Many ALS-linked disease proteins including TDP-43, FUS, EWSR1, TAF15, hnRNPA1, and hnRNPA2 are shown to be recruited into stress granules in dividing cells, and ALS-linked disease mutation of these RBPs can alter stress granule dynamics. Moreover, overexpression of TDP-43 and FUS induce spontaneous formation of stress granules. TDP-43 and FUS are also recruited into stress granules in neurons, although stress granule dynamics are altered between neurons and dividing cells [reviewed in (Wolozin and Ivanov, 2019)]. It is suggested that TDP-43 is required for optimal stress granule dynamics in primary neurons and glia exposed to oxidative stress (Khalfallah et al., 2018). Aged neurons via prolonged culture times have compromised stress granule assembly and decreased TDP-43 expression level (Khalfallah et al., 2018). Additionally, mutant TDP-43 affects stress granule dynamics in motoneuron-like cells (Ding et al., 2021). Similarly, using iPSC-derived motor neurons, it is shown that ALS-causing FUS mutant FUS_P525L_ can alter neuronal stress granule dynamics (Marrone et al., 2018). Many evidence suggest that persistent stress granules provide the environment for aberrant phase transition and pathogenic inclusion formation of RBPs. The role played by stress granules in aberrant phase transition of RBPs will be discussed more detail in the next section.

### Transport Granules and Local Translation

Localized translation of mRNA near neuronal synapses is important for proper neuronal function, thus requiring transport of mRNA from the soma to dendrites and axons (Klann and Dever, 2004; Hirokawa, 2006; Kalinski et al., 2015; Holt et al., 2019). RNP transport granules were first identified in rat cortical neurons (Knowles et al., 1996; Kiebler and Bassell, 2006) and demonstrated to be trafficked by kinesin in mouse hippocampal neurons (Kanai et al., 2004). TDP-43 forms cytoplasmic RNP granules that transport target mRNA to distal neuronal compartments (Alami et al., 2014). TDP-43-containing RNP transport granules also exhibit spatial- and temporal -dependent biophysical properties (Gopal et al., 2017). Additionally, TDP-43 plays an important role in long-distance transport of G-quadruplex-containing mRNA to neurites for their local translation (Alami et al., 2014; Ishiguro et al., 2016; Endo et al., 2018; Thelen and Kye, 2019). ALS-linked TDP-43 mutations lead to decreased interaction with mRNA, reduced RNP granule dynamics and disrupted axonal transport dynamics (Alami et al., 2014; Ishiguro et al., 2016; Gopal et al., 2017; Endo et al., 2018; Thelen and Kye, 2019), implicating TDP-43’s role in local translation. Mislocalized TDP-43 can also affect local translation in axons by promoting G3BP1-positive RNP condensate assembly, consequently inhibiting local protein synthesis (Altman et al., 2021).

Similarly, FUS is involved in neuronal transport granules. As mentioned earlier in this review, FUS was demonstrated to colocalize with RNA granules in dendrites of mouse hippocampal neurons, and the expression of FUS controlled RNA translocation in these dendrites (Fujii and Takumi, 2005). ALS mutations in FUS were shown to disrupt axonal transport and general intra-axonal protein synthesis in cultures of *Xenopus* retinal neurons as well as in mouse sciatic nerve axons *in vivo* (Murakami et al., 2015; Guo et al., 2017; López-Erauskin et al., 2018), thus highlighting the role of FUS in transport granule dynamics.

### RNA Regulation

RNA binding proteins’ function is closely related to the life cycle of RNA, including RNA transcription and post-transcription regulation. Recent studies have proposed a model where transcription is regulated by two phase-separated bio-condensates: initiation condensates and elongation condensates. RNA polymerase II (Pol II) regulates shuttling between these two functional condensates through phosphorylation of its CTD (C-terminal domain) (Guo Y. E. et al., 2019). Importantly, the phase-separated LCDs of FUS, EWSR1, and TAF15 directly bind to the CTD of RNA Pol II *in vitro* and in live cells (Chong et al., 2018). These interactions can be regulated by RNA Pol II CTD phosphorylation (Kwon et al., 2013). Moreover, the transcriptional activity of FUS mutants that harbor mutations in the LCD correlates with their ability of condensate formation (Kwon et al., 2013), indicating the role played by FUS phase separation in transcription regulation.

The role of FUS and TDP-43 phase separation in post-transcription regulation is less clear. Some evidence suggest that modulation of TDP-43 phase separation propensity by mutating conserved glycine residues in the low-complexity domain or by N-terminal phosphomimetic substitution can also modulate the splicing activity of TDP-43 (Wang A. et al., 2018; Conicella et al., 2020). On the other hand, systematic mutation of the TDP-43 IDR have identified a mutant that disrupts phase-separate but maintains its splicing activity (Schmidt et al., 2019). Moreover, studies on phase separation-dependent FUS interactome reveal that compared to the LLPS-specific FUS interactome, factors involved in RNA splicing and mRNA processing were enriched much more significantly in the non-LLPS FUS interactome (Reber et al., 2021). Therefore, further studies dissecting the exact contributions of phase separation to TDP-43 and FUS function in post-transcription regulation are needed.

### DNA Damage Repair Foci

Upon DNA damage, microscopically visible DNA damage repair foci form by recruiting DNA repair factors at the lesions, which activate downstream signaling factors. Recently, it is shown that DNA repair focal assemblies marked by 53BP1 (p53 binding protein 1) are phase-separated compartments formed by the liquid-liquid phase separation of the DNA damage repair factors and non-coding RNA transcribed near the double-strand breaks (Kilic et al., 2019; Pessina et al., 2019). Similar liquid-like behavior has been reported for yeast DNA damage repair foci marked by Rad52 (Oshidari et al., 2020; Miné-Hattab et al., 2021). Interestingly, several RNA-binding proteins involved in neurodegenerative diseases have been implicated in the maintenance of DNA integrity in response to DNA damage, including TDP-43, FUS, hnRNPA1, and Ataxin-2 (Flynn et al., 2011; Skourti-Stathaki and Proudfoot, 2014; Mitra et al., 2019).

Of these RBPs, the role played by FUS is most well-established. FUS involvement in DNA damage repair was first suggested when defects in the repair of DNA damage produced by ionizing radiation in FUS knockout mice was observed (Baechtold et al., 1999; Hicks et al., 2000; Kuroda et al., 2000). Subsequent studies established direct localization of FUS to the DNA damage repair foci, which is dependent on poly(ADP)-ribosyl (PAR) polymerase (PARP) (Mastrocola et al., 2013; Wang et al., 2013; Altmeyer et al., 2015). In addition, FUS is capable of interacting directly with PAR chains through its RGG domain and PAR-binding potently promotes liquid-liquid phase separation of FUS (Altmeyer et al., 2015; Singatulina et al., 2019), indicating the important role played by FUS phase separation in DNA damage repair. Indeed, when the interactome of phase separated-FUS is compared with that of the non-phase separated FUS, it is shown that proteins involved in DNA damage response were almost exclusively detectable together with phase separated FUS (Reber et al., 2021). Moreover, liquid-liquid phase separation of FUS is important for initiation of DNA damage repair as LLPS-deficient variants of FUS affect accumulation of DNA repair factors at sites of laser-induced DNA damage (Levone et al., 2021). Phase separated FUS compartments formed at DNA damage lesions are dynamic and reversible, which dissolve rapidly after PAR removal by PAR glycosylase (Singatulina et al., 2019). Intriguingly, it has been shown that these early phase-separated FUS condensates are incompatible with 53BP1 accumulation and can be replaced by foci formed by 53BP1 (Altmeyer et al., 2015), indicating distinct biomolecular condensates might form at different stages of DNA damage repair to orchestrate the repair process. Not only is FUS phase separation important for DNA damage repair, it is recently shown that impaired DNA damage response signaling by FUS-NLS mutations can lead to aberrant FUS phase transition and aggregate formation in the cytoplasm and neurodegeneration (Naumann et al., 2018). PARG activity, which dissolves FUS foci after stress, has been shown to lead to accumulation of FUS in the cytoplasm of neurons (Naumann et al., 2018). Together, these studies provide an interesting link between DNA damage repair and neurodegenerative diseases.

## Aberrant Phase Transition and Pathological Aggregation of TDP-43 and FUS in ALS/FTD

Functional, phase-separated biomolecular condensates have diverse roles, as discussed in the previous sections in this review. However, dysregulated phase transition can be detrimental. One of the consequences of aberrant phase transition of RNA-binding proteins is the formation of protein aggregates, which is a pathological hallmark in several neurodegenerative diseases, including ALS and FTD. The mechanisms underlying the formation of RBP aggregates and their cellular toxicity is not completely understood. In this section, we will discuss the current understanding of the mechanism of RNA-binding protein aggregation.

### Formation of RNA-Binding Protein Aggregates in ALS/FTD

Although the mechanism of how cytoplasmic aggregates form and cause toxicity in FTD/ALS patients is not yet clear, a link between accumulation of pathological inclusions formed by IDR-containing RBPs and persistent stress granules has been indicated (Bosco et al., 2010; Dormann et al., 2010; Dormann and Haass, 2011; Baron et al., 2013; Vance et al., 2013; Lin et al., 2015; Molliex et al., 2015; Murakami et al., 2015). Dense networks of weak and promiscuous interactions between IDRs and between IDR and RGG domains of RBPs work together synergistically to mediate LLPS (Boeynaems et al., 2018). The natural tendency of these IDR-containing RBPs to engage in promiscuous interactions promotes the formation of functional liquid droplets, such as stress granules, but also renders them prone to aberrant phase transition resulting in solid-like aggregate formation. Indeed, phase-separated stress granules are enriched with aggregation-prone RBPs, such as TDP-43 and FUS. Persistence of stress granules, caused by either failure of granule removal or FTD/ALS-causing mutations in RBPs, has been proposed to provide crucibles for aberrant phase transition that leads to fibrillization of these RBPs (Elbaum-Garfinkle and Brangwynne, 2015; Lin et al., 2015; Molliex et al., 2015; Murakami et al., 2015; Patel et al., 2015). For example, disease-linked mutations in LCDs can increase the solid property and accelerate the liquid-to-solid transition of phase separated RBP condensates (Patel et al., 2015; Conicella et al., 2016; Mackenzie et al., 2017). In addition, disease-linked mutations in the NLS increase the cytoplasmic concentration of the LCD-containing protein and promote stress granule formation. To support this notion, aggregates of these RBPs in patients have been shown to colocalize with other stress granule components, indicating that stress granules may be the sites of disease biogenesis (Dormann et al., 2010; Li et al., 2013). Moreover, increasing numbers of ALS/FTD-causing RBPs are found to be associated with stress granule components, including: hnRNPA1, hnRNPA2/B1, TIA1, Ubiquilin 2, Profilin 1, and Matrin 3 (Andersson et al., 2008; Kim et al., 2013; Sama et al., 2013; Figley et al., 2014; Kamelgarn et al., 2016; Mackenzie et al., 2017; Alexander et al., 2018; Hock et al., 2018; Tada et al., 2018; Zhang et al., 2020). Direct evidence of stress granules as the initial site of RBP aggregates came from engineered light-inducible stress granule developed by Taylor and colleagues. Using this system, they demonstrated repetitive or persistent SG formation directly leads to TDP-43 aggregation (Zhang et al., 2019). However, alternative routes of TDP-43 aggregates formation have also been proposed. For example, it is suggested that stress granules are initially beneficial because highly concentrated RNA and PAR can prevent TDP-43 aberrant phase transition, but prolonged chronic stress leads to growth of insoluble TDP-43 aggregates that persist after stress granule disassembly. Furthermore, it has been shown that TDP-43 aggregates with pathological features of TDP-43 inclusions found in ALS and FTD patients can be formed independent of stress granules (Chen and Cohen, 2019; Mann et al., 2019; Fernandes et al., 2020). Therefore, further investigation is needed to understand the origin of RBP aggregates in ALS/FTD.

### Aggregation of TDP-43 and FUS in ALS/FTD

Regardless of the origin of RBP aggregation, the presence of RBP inclusion bodies are a common pathological hallmark for ALS/FTD. For example, TDP-43 positive inclusion bodies are observed in ∼97% of the ALS cases and ∼45% of all FTLD cases. FUS positive inclusion bodies are observed in ∼1% of the ALS cases and ∼9% of all FTLD cases. Interestingly, TDP-43 and FUS pathology are mutually exclusive (Mackenzie et al., 2007; Neumann et al., 2009; Vance et al., 2009). Hyperphosphorylated TDP-43 aggregates were first identified in post-mortem tissue of familial and sporadic ALS patients (Arai et al., 2006; Neumann et al., 2006), and Ser403/404 and Ser409/410 are the major phosphorylation sites of insoluble TDP-43 in ALS/FTD. It is shown that phosphorylation can alter the aggregation propensity and phase separation property of TDP-43 (Wang A. et al., 2018). A recent study also found phosphomimic mutation S48E disrupts the formation of anisosomes, distinct phase-separated nuclear assemblies (Yu et al., 2021). TDP-43 pathology is also characterized by ubiquitylation and aberrant lysine acetylation (Arai et al., 2006; Neumann et al., 2006; Cohen et al., 2015). Moreover, in addition to full length protein, TDP-43 aggregates in ALS also contain C-terminal truncations which are the result of splicing defects and proteolytic cleavage. These ∼25–35 kDa C-terminal fragments (CTFs) contain the PrLD, which harbors most of the ALS-associated TDP-43 mutations and phosphorylation sites (Lattante et al., 2013). Several of these mutations show enhanced aggregation propensity when purified and increased cytotoxicity when expressed in yeast and neurons (Johnson et al., 2009; Guo et al., 2011). The CTFs are highly toxic when expressed in neurons and in isolation, purified C-terminal TDP-43 truncation forms fibrils with different morphology compared to full length TDP-43 aggregates (McGurk et al., 2018b).

In FTD patients, FUS inclusions are characterized by hypomethylation of the RGG domain, which enhances the phase separation propensity and stress granule recruitment of FUS (Hofweber et al., 2018). On the other hand, FUS aggregates in ALS are characterized by arginine methylation in the RGG domain that reduces binding to its nuclear import receptor Kapβ2. Indeed, while Kapβ2 was abundant in FUS-immunopositive inclusions in FTD-FUS, it was not observed in FUS inclusions in ALS patients (Brelstaff et al., 2011; Troakes et al., 2013). Another difference between FUS aggregates in ALS and FTD is coaggregation with other FET family proteins EWSR1 and TAF15, which was observed only in FTD patients (Neumann et al., 2012), indicating different pathomechanism of ALS and FTD. Most of the ALS/FTD-causing mutations are located in the PrLD or PY-NLS of FUS (Harrison and Shorter, 2017). While mutations in the PrLD can accelerate aberrant phase transition and enhance aggregation propensity of FUS, mutations in the PY-NLS do not change the aggregation property of FUS (Sun et al., 2011; Patel et al., 2015). Therefore, mutations in the PY-NLS might cause FUS aggregation through a different mechanism. Indeed, mutations in FUS PY-NLS disrupt its binding to Kapβ2 and result in cytoplasmic accumulation of FUS(Hofweber et al., 2018). Increased cytoplasmic FUS concentration leads to enhanced recruitment to stress granules. Furthermore, it has been shown that Kapβ2 can function as chaperone and protein disaggregase for cytoplasmic FUS (Guo et al., 2018; Hofweber et al., 2018; Yoshizawa et al., 2018). Mutations in PY-NLS reduces Kapβ2’s activity in preventing and reversing FUS aggregation and aberrant phase transition, contributing to the accumulation of cytoplasmic FUS aggregates (Guo et al., 2018).

### Spreading of Protein Aggregates in ALS/FTD

A characteristic feature of ALS and FTD is the spreading of symptoms from its original onsite to nearby contiguous anatomical regions in the CNS in a spatiotemporal manner (Ravits et al., 2007; Ravits and La Spada, 2009). The spread of symptoms in ALS/FTD indicates that propagating agents might be present in these diseases. In fact, it has been suggested that protein misfolding and aggregation can spread in a prion-like mechanism in neurodegenerative diseases (Brundin et al., 2010). Prions are self-replicating infectious protein conformers that can be used as templates to seed the folding of soluble proteins comprised of the same amino acid sequence. While the prions formed by mammalian prion protein (PrP) often cause deadly neurodegenerative diseases, prions in yeast are often benign (March et al., 2016). Interestingly, many human RBP aggregates in ALS harbor low complexity domains similar in amino acid composition to yeast PrLDs (prion-like domains; PrLDs) (March et al., 2016). Indeed, both TDP-43 and FUS have PrLDs and multiple lines of evidence suggest that a prion-like propagation mechanism is active in ALS and FTD. For example, addition of preformed recombinant TDP-43 fibrils in neuronal cell lines can seed aggregation of both over-expressed and endogenous TDP-43 (Furukawa et al., 2011; Nonaka et al., 2013; Gasset-Rosa et al., 2019). Interestingly, preformed recombinant FUS fibrils could also seed TDP-43 aggregation when added into cultured cells (Gasset-Rosa et al., 2019). Another characteristic of prion-like activity is cell-to-cell transmission of misfolded protein, which has also been documented for TDP-43. When insoluble TDP-43 extracted from ALS/FTD patients was introduced into TDP-43-expressing neuronal cells, TDP-43 aggregates with pathological hallmarks of patient TDP-43 inclusion (i.e., phosphorylated and ubiquitinated) were induced in a seed-dependent manner (Nonaka et al., 2013). Furthermore, when FTD brain extracts were injected into transgenic mouse model expressing human TDP-43 with mutated NLS signal, seeded TDP-43 pathology has been shown to spread cell-to-cell from one brain region to another (Porta et al., 2018). Moreover, a recent study showed that cryo-EM structure of TDP-43 filaments extracted from both frontal and motor cortices of ALS/FTD patients share the same double-spiral fold, consistent with the temporospatial spread of TDP-43 aggregates (Arseni et al., 2021). Recently, Laferrière et al. showed that TDP-43 aggregates extracted from different FTD disease subtypes exhibit distinct biochemical and morphological features, and the biochemical and morphological differences of different TDP-43 conformations were associated with differential seeding and neurotoxic potential (Laferrière et al., 2019). This study provides strong evidence for the existence of TDP-43 prion-strains and it would be interesting to see whether different TDP-43 conformations can propagate *in vivo*.

## Methods to Reverse Protein Phase Separation and Aggregation in Neurons

Currently, the FDA has approved two generic drugs for the treatment of ALS, Riluzole and Edaravone, which have demonstrated increased patient survival (Bensimon et al., 1994; Abe et al., 2017; Rothstein, 2017; Fang et al., 2018; Oskarsson et al., 2018). Another drug, Nuedexta (dextromethorphan HBr and quinidine sulfate), is available as an adjunct for ALS patients with pseudobulbar affect (Yang and Deeks, 2015; Smith et al., 2017). While these treatments offer great benefit to the patient, they do not present a cure nor do they address the underlying proteinopathies involved in ALS.

A promising therapeutic strategy targeting the disease-associated proteinopathies are antisense oligonucleotides (ASOs), which work by targeting mRNA and promoting rnase H-mediated degradation. Currently, multiple ASOs are in development and clinical trials [further reviewed in (Ly and Miller, 2018)]. Most target SOD1 or C9orf72, but recently, others are being developed against RBPs such as FUS. Jacifusen (ION363, ionis Pharmaceuticals) is one such ASO that targets FUS by preventing the translation of mutated *FUS* mRNA and is currently in phase 3 clinical trials (NCT04768972).

**FIGURE 3:**
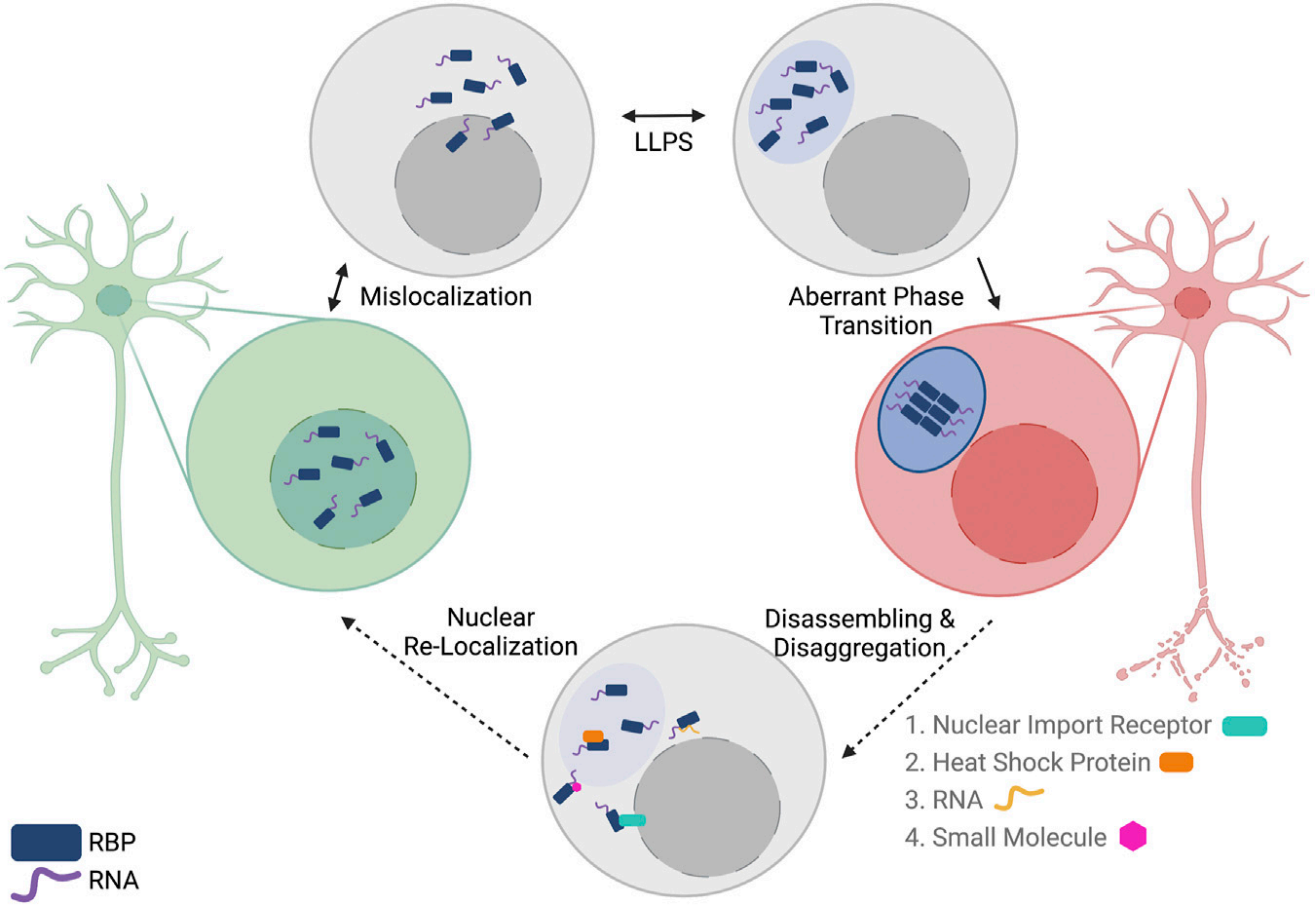
Reversing aberrant phase transition of nuclear RNA-binding proteins (RBPs) as a potential therapeutic option for patients with ALS/FTD. In ALS/FTD, nuclear RBPs are first mislocalized to the cytoplasm. In the cytoplasm, RBPs can undergo reversible liquid-liquid phase separation (LLPS) to form liquid condensates. In some cases, RBPs undergo aberrant phase transition into a solid-like phase where the RBPs can assemble into fibrils. Multiple methods of disassembling and disaggregating proteins are proposed, including the use of proteins, RNAs, and small molecules. These biomolecules could potentially reverse aberrant phase transition and allow for subsequent nuclear localization of the RBPs by canonical mechanisms. Created with BioRender.com.

The proteinopathies observed in ALS and the association between LLPS and neurodegenerative diseases has led many to seek methods to reverse phase separation of RBPs in the hopes of developing new therapeutic options for patients with ALS. Recently, a variety of disaggregases have been described that range from other protein interactors to RNAs to small molecule inhibitors (Figure 3). The following represent potential therapeutic strategies that could prevent and/or reverse protein aggregation of ALS-associated RBPs. It is important to note that these methods have not been tested in higher-order model systems, but given their results *in vitro*, more research should focus on these potential strategies for RBP disaggregation.

### Nuclear Import Receptors

Cytoplasmic mislocalization and subsequent aggregation of nuclear RBPs are pathological hallmarks of ALS and FTD. As discussed in the previous section, in the cytoplasm, these proteins are recruited into stress granules, and under pathological conditions, can undergo aberrant phase transition and mature into aggregates. There is a vast wealth of evidence demonstrating the functional cytoplasmic localization of the aforementioned nuclear RBPs; however, these proteins cannot accumulate in the cytoplasm and must be imported back to the nucleus. The karyopherin family of nuclear import and export receptors are responsible for most protein trafficking between the nucleus and the cytoplasm. Most proteins are exported from the nucleus through Exportin-1/CRM1; however, both TDP-43 and FUS are localized to the cytoplasm independent from this export receptor via passive diffusion (Ederle et al., 2018; Pinarbasi et al., 2018). While it is not yet understood why TDP-43 and FUS are exported via passive mechanisms, their subsequent nuclear import is well-characterized.

TDP-43 contains a bipartite classical nuclear localization signal near its N-terminus that facilitates its recognition by the importin a-karyopherin b complex. In this mechanism, TDP-43 NLS is recognized by importin a, which acts as an adaptor for karyopherin b1 recognition (Nishimura et al., 2010; Marfori et al., 2011). Using the established RAN gradient, this protein complex is able to traverse the nuclear pore complex and deposit TDP-43 in the nucleus. In contrast, FUS nuclear import is mediated by its non-classical, bipartite C-terminal proline tyrosine nuclear localization signal (PY-NLS) spanning residues 498 to 526. This domain interacts with C-terminal arch of karyopherin b2 (Kapb2) (Cansizoglu and Chook, 2007; Zhang and Chook, 2012). Direct binding of FUS PY-NLS to Kapb2 facilitates FUS transport through the nuclear pore and release into the nucleus upon Kapb2-RAN-GTP binding. Of note for future studies, the position and type of tag affects the cytonuclear localization of both TDP-43 and FUS. For example, when TDP-43 is tagged with tdTomato, it remains nuclear whereas when it is FLAG-tagged, it has increased cytoplasmic localization (Pinarbasi et al., 2018).

Recently, multiple groups have demonstrated that nuclear import receptors can prevent and reverse aberrant phase transition of TDP-43, FUS, hnRNPA1 and hnRNPA2/B1 (Guo et al., 2018; Hofweber et al., 2018; Qamar et al., 2018; Yoshizawa et al., 2018; Guo L. et al., 2019; Niaki et al., 2020). It is widely known that the karyopherin family have redundant functions and multiple transport receptors can bind the same cargo protein. For example, a recent study demonstrated that FUS can form stable complexes with additional import receptors, such as transportin-3, importin b, and importin 7, and these interactions reduced FUS stress granule recruitment in HeLa cells (Baade et al., 2021). Since they are in the same family as Kapb2, it is possible that these newly-identified transport receptors may be new targets to explore.

### Heat Shock Proteins

Protein aggregation is associated with general protein misfolding and the natural mediators of misfolding in the cell are molecular chaperones. A subset of molecular chaperones are heat shock proteins (Hsps), which are normally upregulated in response to stress stimuli and have demonstrated the ability to regulate phase separation of RBPs. One recent example is HspB8 ability to regulate FUS phase separation, which was published by Boczek et al. earlier this year (Boczek et al., 2021). They showed that HspB8 can be recruited into FUS condensates and prevents aberrant phase transition of FUS condensates by preventing FUS RRM unfolding. Specifically, this chaperoning is seemingly accomplished through interactions of its alpha-crystallin domain with the FUS RRM. Another example comes from Mateju et al., which implicates Hsp70 in regulating SG maturation and aberrant phase transition (Mateju et al., 2017). Specifically, Yu et al. recently demonstrated Hsp70 can recruit cytoplasmic, RNA-free TDP-43 into condensates with liquid-inside-liquid structure, termed anisosomes, in neuron-like cells and iPSC-derived motor neurons (Yu et al., 2021). Further, they demonstrate that Hsp70 prevents TDP-43 anisosome gelation in U2OS cells in an ATP-dependent manner (Yu et al., 2021). In addition to metazoan heat shock proteins, Hsp104 is a yeast-derived chaperone with observed disaggregase activity. While wild-type Hsp104 is unable to disaggregate TDP-43 or FUS, multiple studies from the Shorter lab demonstrate chaperone capabilities of Hsp104 variants in reversing aggregation of a-Synuclein, TDP-43 and FUS in multiple *in vitro* models (Jackrel et al., 2014; Tariq et al., 2019), suggesting Hsp104 potential as a disaggregase for RBPs involved in ALS and FTD. There are many other heat shock proteins present in neuronal populations, and their potential as RBP disaggregases are further discussed by Shorter (Shorter, 2017).

### RNA

A natural binding partner of RBPs are RNAs, which interact with RBPs through a variety of sequence and structural motifs. The nuclear RBPs associated with ALS and FTD have varying specificity for RNAs, but it has been demonstrated by multiple investigators that RNA treatment can prevent and reverse phase separation of these proteins. RNAs by nature are uniquely primed for pharmacological development, thus RNAs may represent another therapeutic avenue worth further exploration.

TDP-43 often interacts with RNAs, and previous investigations have shown that TDP-43 favors GU-rich RNA sequences (Polymenidou et al., 2011; Tollervey et al., 2011; Lukavsky et al., 2013). Specifically, TDP-43 seems to recognize GU-rich RNAs through its RRM domains to oligomerize and that these repeats prevent TDP-43 phase separation *in vitro* (Rengifo-Gonzalez et al., 2021). Recently, investigators have also shown that RNA influences the phase separation of TDP-43 *in vitro* and that increasing RNA concentration increased the solubility of TDP-43 (Maharana et al., 2018). Others have also demonstrated that specific RNA sequences can affect the solubility of TDP-43. One such sequence is Clip_34, which comes from the 3’ UTR of TDP-43 and was identified to have high affinity for TDP-43 in a CLIP experiment in HEK293 cells (Ayala et al., 2008; Bhardwaj et al., 2013). Additionally, Clip_34 was shown to prevent phase separation and aberrant phase transition of TDP-43 in HEK293 cells and increase survival and nuclear localization in neuronal cell lines (Mann et al., 2019). In all, RNAs seem capable of preventing and reversing phase separation of TDP-43 and could be further developed into potential therapeutics.

In line with its functions in mRNA transport, local translation and splicing, FUS readily interacts with RNAs. Previous studies have highlighted the complexity of FUS-RNA binding interactions, and we now understand that FUS can recognize a variety of RNA sequence and structural motifs with differing affinities (Iko et al., 2004; Wang et al., 2015; Loughlin et al., 2019). One such sequence motif is GGUG, and previous work demonstrates that FUS ZnF binds GGUG-containing RNA with a Kd of 10 µM and that the GGUG motif recognition appears to be important for pre-mRNA splicing by FUS (Iko et al., 2004). Daigle et al. demonstrated important functional consequences of FUS-RNA interactions including cytoplasmic mislocalization and stress granule recruitment, suggesting RNA can regulate FUS in neurons (Daigle et al., 2013). These earlier studies demonstrated that FUS-RNA binding is crucial for FUS function, and many investigators have since explored the effect of RNA binding on FUS phase separation. *In vitro* phase separation of FUS is increased by RNA when mass ratio of RNA to FUS is sub-stoichiometric, but phase separation decreases when this ratio is increased to stoichiometric levels, suggesting a concentration-dependent effect of RNA on FUS phase separation (Burke et al., 2015). This seems to play a role in the solubility of FUS in the cytoplasm and nucleus as Maharana et al. demonstrates that FUS will form foci in HeLa nuclei after adding rnase (Maharana et al., 2018). This study also demonstrated decreased FUS phase separation with increasing total RNA concentration *in vitro*, and RNA seems to prevent or slow-down aberrant phase transition *in vitro* (Maharana et al., 2018). Taking these findings into consideration, RNA could potentially be used as a disaggregase against FUS.

### Other Potential Regulators of ALS-Associated Protein LLPS and Aggregation

While the three strategies mentioned above are promising methods to reverse phase separation of ALS-associated nuclear RBPs, other candidate approaches have demonstrated a similar effect on LLPS. Much of the following strategies use small molecules that are already in use for treatment of other diseases, mainly cancer, and are extensively reviewed by Brown and colleagues (Brown et al., 2020). This is advantageous because these molecules are typically FDA-approved drugs, so much of the toxicity data in humans and their general acquisition is widely available.

#### Small Molecule Inhibitors

Other researchers have screened for small molecules that may disrupt the weak interactions between low-complexity domains in RBPs associated with ALS. The goal of this strategy would be to prevent phase separation of RBPs and a successful mechanism seems to rely on the ability of the small molecule to interact with RNA and prevent RNA-binding to RBPs. One recent example comes from Fang et al. where they identified multiple small molecules that disrupt stress granule formation in HEK293T cells. They validated these molecules in iPS-derived motor neurons and found that planar compounds, such as mitoxantrone, slowed SG growth and interacted with RNA such that TDP-43 and hnRNPA2/B1 were decreased in SG fractions from neural progenitor cells (Fang et al., 2019).

#### PARP Inhibitors

Poly(ADP-ribose) polymerase (PARP) is an enzyme that is implicated in enhancing phase separation of RBPs. For example, PARylation is a PTM found on RBPs, such as FUS and hnRNPA1, and multiple investigators recently demonstrated that PAR modifications enhance phase separation of these proteins (McGurk et al., 2018a; Duan et al., 2019). This modification is attached to proteins by PARP, specifically PARP1, and multiple studies have demonstrated that the use of PARPi (PARP inhibitors), such as olaparib, suppresses their phase separation *in vitro* (Altmeyer et al., 2015; Duan et al., 2019).

#### Kinase Inhibitors

Kinases are the enzymes responsible for phosphorylating proteins, thus inhibiting kinases could decrease the phosphorylation and subsequent phase separation of RBPs. Small molecule kinase inhibitors have been previously developed and are commonly used in cancer treatment. Thus, kinase inhibitors represent a promising drug class that warrants further investigation. As mentioned earlier in this review, modulating post-translational modification of RBPs can prevent and reverse their phase separation. One critical modification is phosphorylation of serines, threonines, and tyrosines, which are commonly present in the PrLD of RBPs. A promising example is the use of kinase inhibitors to target TDP-43 aggregation. Protein casein kinase-1a (CK-1a) was discovered as a kinase associated with TDP-43 phosphorylation and aggregation (Nonaka et al., 2016; Hicks et al., 2020). A recent report demonstrated decreased TDP-43 phosphorylation after treatment with a CK-1a inhibitor, IGS-2.7, and demonstrated CK-1a inhibition was correlated with increased motor neuron viability (Martinez-Gonzalez et al., 2020). Other kinases, including CDC7 and TTBK, are also implicated with TDP-43 phosphorylation and may represent alternate targets (Liachko et al., 2013; Liachko et al., 2014).

#### Pur-Alpha

The Pur protein family is a group of highly-conserved proteins that have nucleotide-binding abilities. Specifically, they are ubiquitous transcriptional activators with high sequence specificity for purine-rich single-stranded DNA and RNA. While Pur-alpha (Pura) is expressed in all mammalian tissues, it has important implications in the CNS. For example, deletions of the *PURA* gene cause PURA syndrome, a neurodevelopmental disorder. There is also evidence demonstrating Pur-alpha can associate with RNAs including *NEAT1* and *C9orf72* repeat expansions, and it can be incorporated into stress granules [further reviewed in (Molitor et al., 2021)]. Recently, Daigle et al. identified Pur-alpha in stress granules derived from FUS-ALS patients and, upon shRNA-mediated knockdown in HEK293T cells, prevented stress granule formation, suggesting Pur-alpha may be able to regulate FUS LLPS (Daigle et al., 2016). Additionally, this study demonstrated that Pur-alpha co-expression can mitigate FUS toxicity and rescue dendritic loss in rat primary motor neurons by preventing cytoplasmic mislocalization of the mutant FUS (Daigle et al., 2016).

## Concluding Remarks

Investigations into the physiological and pathological consequences of protein phase separation has deepened our understanding of why proteins undergo LLPS and how this phenomenon may go awry in disease; however, there remains much to be discovered about LLPS and aberrant phase transition. For example, the consequences of RBP phase separation does not seem to be solely beneficial or detrimental. As mentioned in the Transport Granules section, TDP-43 phase separation is important to facilitate local translation of mRNA at dendrites and axons through transport granules. However, a recent article by Altman et al. suggests that TDP-43 phase separation causes decreased mitochondrial protein translation in axons and at the neuromuscular junction (Altman et al., 2021), thus adding nuance to the functional role of phase-separated TDP-43 in neurons and calls for further investigation.

Another key question relates to the role of stress granules in aberrant phase transition and the mechanism(s) driving this transformation and subsequent protein aggregation. Stress granule-mediated protein aggregation has been widely debated, as multiple groups have recently demonstrated that TDP-43 aggregation does not occur solely through stress granule formation or maturation (Gasset-Rosa et al., 2019; Fernandes et al., 2020; Watanabe et al., 2020); however, other groups have observed RBP aggregation mediated by stress granules [further reviewed in (Baradaran-Heravi et al., 2020)]. These studies highlight the possibility that multiple aggregation pathways may contribute to the pathological protein aggregates observed in ALS/FTD patients.

Liquid-liquid phase separation of TDP-43 and FUS is essential for numerous cellular functions, yet dysregulated phase separation of these proteins can lead to protein aggregation and be detrimental to neuronal cells (Figures 2, 3). The pathological consequences of protein aggregation are widely undiscovered and has led many to investigate whether protein aggregates contribute to or are a consequence of ALS pathology [further discussed in relation to TDP-43 in (Hergesheimer et al., 2019)]. However, strategies to prevent and reverse aberrant protein assembly have the potential to prevent the spreading of aberrant protein phase in neurodegenerative diseases such as ALS and FTD (Figure 3). With deeper understanding of factors that mediate phase separation of TDP-43 and FUS, as well as the mechanism of how their phase separation contributes to their cellular function, more targeted approaches can be designed to restore the functional protein phase of TDP-43 and FUS.

## Abbreviations

RBP, RNA-binding protein; LLPS, liquid-liquid phase separation; SG, stress granule; LCD, low-complexity domain; PrLD, Prion-like domain; NTD, N-terminal domain; CTD, C-terminal domain; RGG, arginine-glycine-glycine domain.
